# The Positive Impact of a Chemotherapeutic Approach on Relapse Ependymoma or WHO Grade III Anaplastic Ependymoma: Report of a Rare Case

**DOI:** 10.7759/cureus.32534

**Published:** 2022-12-14

**Authors:** Abhyuday Meghe, Mayur B Wanjari, Gajanan N Umalkar, Ranjana Sharma

**Affiliations:** 1 Department of Internal Medicine, Acharya Vinoba Bhave Rural Hospital, Jawaharlal Nehru Medical College, Datta Meghe Institute of Medical Sciences, Wardha, IND; 2 Department of Emergency Medicine, Acharya Vinoba Bhave Rural Hospital, Jawaharlal Nehru Medical College, Datta Meghe Institute of Medical Sciences, Wardha, IND; 3 Department of Medical Surgical Nursing, Srimati Radhikabai Meghe Memorial College Of Nursing, Datta Meghe Institute of Medical Sciences, Wardha, IND

**Keywords:** recurrent ependymoma, reirradiation, toxicity, tumor, relapsed ependymoma

## Abstract

Children with recurrent ependymoma have a poor prognosis. Reirradiation has been proposed as an effective treatment for relapsed ependymoma. In this report, we present the case of a 14-year-old male child with a World Health Organization (WHO) grade III relapse ependymoma, emphasizing the imaging feature that helps differentiate the relapse ependymoma, which is a rarer condition in children. Being able to determine this tumor by its imaging appearance is important to risk stratify patient management decisions. The survival rate of ependymoma is usually five years, but in this case, we present a 14-year-old male child alive with reirradiation and chemotherapy management. The prognosis of the patient after undergoing treatment was good.

## Introduction

Globally, in children, the third most common type of brain tumor is ependymomas. Most tumors (about 90%) occur in the intracranial portion, and about 80% are found in the posterior fossa region [1]. More than half of the cases of this tumor are reported in children aged less than 5 years. The five-year overall survival is 56% to 85%, and the progression-free survival is 38% to 74%, suggestive of the poor prognosis of this disease [2]. Due to the development of resistance to several chemotherapeutic drugs, adjuvant radiation is one of the most important treatment modalities in children aged over 1.5 years [3].

Current treatment approaches for ependymoma have not been standardized. However, reirradiation has demonstrated promising clinical outcomes as compared to chemotherapy. In some cases, this method can be curative in patients where the tumor mass can be completely resected [4]. The occurrence of relapse ependymoma in children is rare and mainly occurs at the age of 5 years with high mortality, but in our case, this occurs at the age of 14 years with a good prognosis reported.

## Case presentation

A 14-year-old male adolescent had presented to the emergency department with headache and radicular pain in limbs for the last four months. On investigation, the patient had been diagnosed with the World Health Organization (WHO) grade III ependymoma. On physical examination, the patient oral intake was poor; the patient required nasogastric tube feeding, but his parents were unwilling to do the procedure. On neurological examination, the Glasgow Coma Scale (GCS) score was 7. He underwent a left parietal craniotomy with a gross total resection in August 2021. The patient revisited in November 2021 with headache and pain in lower limbs; after undergoing MRI, the patient was diagnosed with relapsed ependymoma.

Reirradiation and chemotherapy treatment modalities were given to the patient after the patient's diagnosis of ependymoma relapse; the physician advised chemotherapy. The patient was given curative chemotherapy with intensity-modulated radiation therapy (IMRT) technique. The patient was treated with 33 to 59.4 Gy doses with 6 MV photon energy. The two chemotherapy regimens comprised vincristine injection of 1.5 mg in 10 mL over 10 minutes, carboplatin injection of 560 mg in 500 mL over three hours, and etoposide injection of 150 mg in 400 mL over two hours.

**Figure 1:**
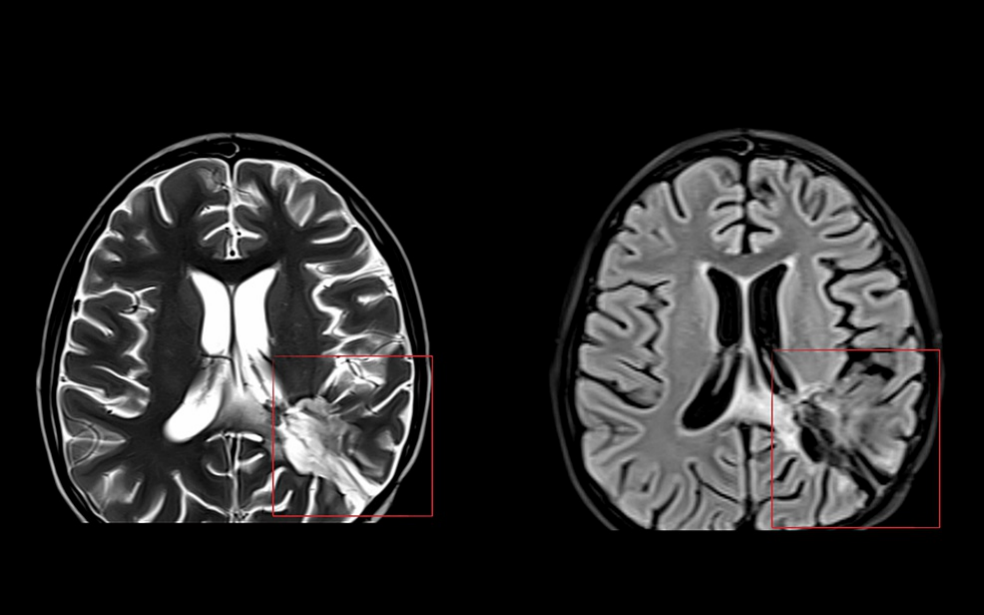
A stable thin rim of the subdural collection was seen along the left temporal and parietal lobes.

On radiological investigation, the MRI of the occurrence of relapsed ependymoma revealed a postoperative resection cavity in the left parietal-temporal lobe, following CSF signal intensity on all pulse sequences. There is peri cavitary nonenhancing T2W/Flair hyperintensity in the left parietal-temporal lobe extending into the splenium of the corpus callosum. There is a marginal decrease of 0.2 cm × 0.2 cm × 0.2 cm in the previously examined enhancing nodular lesion seen in the choroid plexus of the atrium of the lateral ventricle, which was previously 1.2 cm × 1 cm × 0.9 cm. There was no significant change in the small enhancing nodular lesion size in the right CP angle cistern abutting the right inferior cerebellar peduncle, cerebellum posteriorly, 7th-8th nerve complex, and petrous temporal bone anteriorly, measuring 1.1 cm × 1 cm (earlier measurement 1.2 cm × 1 cm; Figure 1). Fourteen days after the completion of chemotherapy, the patient visited for a follow-up. The outcome after the therapeutic intervention had a good prognosis, and the GCS score was 9 when this case was reported.

## Discussion

The WHO has proposed a grading system that classifies subependymoma, ependymoma, and anaplastic ependymoma as grade I, grade II, and grade III. Low-grade ependymoma frequently occurs in infratentorial areas, while high-grade anaplastic ependymoma is majorly found in the cerebral hemisphere [5]. Prognostic factors include the resection area, approach of radiation therapy, chemotherapy, histopathological grade, location, etc. Ependymoma of grade III, as expected, has a worse prognosis as compared to the low grade [6].

Most evidence states that chemotherapeutic intervention in ependymoma is a highly controversial issue. The therapy did not affect the outcome in children with ependymoma. Chemotherapy is used as an adjuvant treatment during the first line of treatment to increase respectability or avoid radiotherapy [7]. However, radiotherapy is widely accepted as an adjuvant therapeutic strategy for patients with ependymoma [8]. The studies show that conventional chemotherapeutic approaches fail to demonstrate clinically significant outcomes in patients with relapse ependymoma. Several chemo-drugs, such as etoposide, cyclophosphamide, and carboplatin, have been given singly or in combination. But no benefit was observed with this approach considering the overall survival during relapse [8,9].

In this case, an anaplastic ependymoma tumor arose in the right anteroinferior cerebellar parenchyma, which was associated with previously reported manifestation. The fascinating characteristics of our case are the relapse of ependymomas grade III after the surgical intervention that was left parietal craniotomy with a gross total resection on August 2021. An MRI was performed on the patient, which indicated a surgical resection cavity in the left parietal-temporal lobe.

Surgical treatment alone, as well as postoperative chemotherapy and radiotherapy, was also reported. In cases with incomplete surgical resection, chemotherapy may be recommended as one of the treatment plans. In our patient, curative chemotherapy with intensity-modulated radiation therapy was given [10]. The patient who was affected by relapse ependymoma had an unpredictable clinical course, which might even lead to a patient's death when a minimal therapeutic option is available, thereby making the management of such a patient very difficult [10].

Relapsed ependymoma has a poor prognosis, with an indolent chronic course, recurrent relapses, and death in around 90% of patients. In the initial condition, a complete surgical resection procedure is the most effective outcome. On a case-by-case basis, re-irradiation therapy may be considered. In the case of relapsed ependymomas and subsequent treatment, 25% of patients had a five-year survival rate. This suggested that this tumor is highly aggressive and has an inferior outcome [10].

## Conclusions

Reirradiation therapy proves to be an effective treatment strategy for recurrent ependymoma cases, which can alter the clinical history of such patients. Conversely, there may exist any other concomitant associations such as neurocognitive toxicity. Further, to investigate the probability of late recurrence, i.e., tumors induced due to secondary radiation, additional follow-up of these patients becomes imperative. It will also aid in assessing the long-term functional outcomes in these patients.
